# High lymphatic vessel density and presence of lymphovascular invasion both predict poor prognosis in breast cancer

**DOI:** 10.1186/s12885-017-3338-x

**Published:** 2017-05-17

**Authors:** Song Zhang, Dong Zhang, Mingfu Gong, Li Wen, Cuiwei Liao, Liguang Zou

**Affiliations:** Department of Radiology, Xinqiao Hospital, Third Military Medical University, Chongqing, 400037 China

**Keywords:** Lymphatic vessel density, Lymphovascular invasion, Disease-free survival, Overall survival, Breast cancer

## Abstract

**Background:**

Lymphatic vessel density and lymphovascular invasion are commonly assessed to identify the clinicopathological outcomes in breast cancer. However, the prognostic values of them on patients’ survival are still uncertain.

**Methods:**

Databases of PubMed, Embase, and Web of Science were searched from inception up to 30 June 2016. The hazard ratio with its 95% confidence interval was used to determine the prognostic effects of lymphatic vessel density and lymphovascular invasion on disease-free survival and overall survival in breast cancer.

**Results:**

Nineteen studies, involving 4215 participants, were included in this study. With the combination of the results of lymphatic vessel density, the pooled hazard ratios and 95% confidence intervals were 2.02 (1.69–2.40) for disease-free survival and 2.88 (2.07–4.01) for overall survival, respectively. For lymphovascular invasion study, the pooled hazard ratios and 95% confidence intervals were 1.81 (1.57–2.08) for disease-free survival and 1.64 (1.43–1.87) for overall survival, respectively. In addition, 29.56% (827/2798) of participants presented with lymphovascular invasion in total.

**Conclusions:**

Our study demonstrates that lymphatic vessel density and lymphovascular invasion can predict poor prognosis in breast cancer. Standardized assessments of lymphatic vessel density and lymphovascular invasion are needed.

**Electronic supplementary material:**

The online version of this article (doi:10.1186/s12885-017-3338-x) contains supplementary material, which is available to authorized users.

## Background

Breast cancer is one of the most common malignant tumors in females. Prognostic factors are helpful in clinical management and have the potential to improve the disease-free survival (DFS) and overall survival (OS) in breast cancer [1]. Several independent risk factors for survival have been identified, including tumor size, histological grade, nodal status, hormone receptor status, and HER-2 status [2, 3]. However, these risk factors are insufficient to fully determine an individual’s prognosis. More risk factors are needed to be explored.

Lymphatic vessel was formerly considered as a passive participant in tumor metastasis and regarded mainly as a transportation channel for tumor cells. Now, it appears that lymphatic vessel provides a safe route for tumor cells dissemination, because of the discontinuous structure of the lymphatic basement membrane, an ultraminiature shear stress, and a high concentration of hyaluronic acid [4]. Even so, it is still uncertain that whether the high lymphatic vessel density is a necessary condition for tumor metastasis. Many studies have demonstrated the unfavorable prognostic value of lymphatic vessel density in primary breast cancer [5, 6]. However, Zhang et al. [7] showed that lymphovascular invasion, but not lymphangiogenesis, was correlated with lymph node metastasis and poor prognosis in young breast cancer patients. Other studies found that the lymphatic vessel density in the lymph node metastasis negative group even was higher than that of the positive group in primary breast cancer [8, 9]. Therefore, a meta-analysis study is needed to pool the results to clarify the prognostic value of lymphatic vessel density in breast cancer.

Lymphatic metastasis contains a series of sequential processes, such as tumor associated lymphangiogenesis, lymphovascular invasion, implantation of cancer cells in regional lymph nodes, and proliferation of micrometastasis in distant organs [10]. Lymphovascular invasion, infiltration of tumor cells into lymphatic vessels, represents a high invasion feature of breast tumor cells. Determined by hematoxylin and eosin (H&E) staining in past time, lymphovascular invasion was widely investigated and showed a correlation with the clinicopathological outcomes of breast cancer [11, 12]. At present, lymphatic vessels can be distinguished from blood vessels or retraction artifacts. Thus, using immunohistochemical staining, many studies have updated the investigation of the prognostic value of lymphovascular invasion [13, 14].

With the identification of specific markers of lymphatic vessels, such as podoplanin/D2–40, LYVE-1, Prox-1 and VEGFR-3, many studies have demonstrated the importance of lymphatic system in tumor metastasis [9]. Therefore, we conducted a meta-analysis study not only to estimate the effect of lymphatic microvessel density on patients’ survival, but also to update and re-estimate the prognostic value of lymphovascular invasion in breast cancer.

## Methods

### Literature search

Databases of PubMed, Embase and Web of Science were searched from inception up to 30 June 2016 by two independent observers. The following Medical Subject Heading (MeSH) terms or keywords were used: “breast cancer OR breast carcinoma OR breast neoplasms” AND “lymphatic vessel density OR lymphatic microvessel density OR LVD OR LMVD OR lymphangiogenesis OR lymphovascular invasion OR lymphatic vessel invasion OR lymphatic invasion OR LVI” AND “prognostic OR prognosis OR survival”. All abstracts mentioned the prognostic values of lymphatic vessel density or lymphovascular invasion, no matter prospective or retrospective, were selected for further consideration.

### Inclusion criteria

The studies met the following criteria could be included: (1) treated with the patients with primary breast cancer only, instead of the patients who were previously diagnosed with other diseases; (2) published as a full paper, by no means of review papers, case reports, meeting abstracts, or animal researches; (3) determined lymphovascular invasion presence by immunohistochemical staining, rather than hematoxylin and eosin (H&E) staining. Two independent authors followed the inclusion criteria to review the publications. When two or more articles reported duplicating data, only the study with the most recent data, or the largest dataset was included.

### Data extraction

The final eligible studies were conducted the data extraction with a standardized form. The data retrieved from the papers included the first author’s name, year, country, number of the patients (size), age, antibody and its dilution, follow-up period, cutoff value of lymphatic vessel density, detection rate of lymphovascular invasion, and the results of DFS and OS. The key components of designs were used to estimate the quality of primary studies, based on the criteria of the Newcastle-Ottawa Quality Assessment scale (NOS) [15].

### Statistical analysis

The extracted data were analyzed by using STATA software version 12.0 (STATA Corporation, College Station, Texas, USA). We evaluated the impacts of lymphatic vessel density, lymphovascular invasion on survival by pooling the hazard ratio (HR) results. HR values and their corresponding 95% confidence intervals (95% CIs) were obtained by the methods as previously reported [16]. In method one, the HRs were directly acquired from the publications. In method two, the HRs were calculated from the total number of events and its *P* value, or from the O-E statistic (difference between numbers of observed and expected events) and its variance. In method three, the survival rate at the end point of the survival curve was extracted to reconstruct the estimated HR and its variance, with the assumption that the rate of patients censored was constant during the follow-up period. The estimated HR values were combined into an overall HR value using Peto’s method. Homogeneity test was performed with Q statistic and I^2^ statistic. A random-effects model or, in the absence of heterogeneity, a fixed-effects model was applied to combine the HR values. An observed HR > 1 represented a worse survival for the group with a high lymphatic vessel density or presence of lymphovascular invasion. *P* < 0.05 and I^2^ > 50% were considered as statistically significant. Publication bias was evaluated using a funnel plot of Egger’s test.

## Results

### Study selection process

**Fig. 1:**
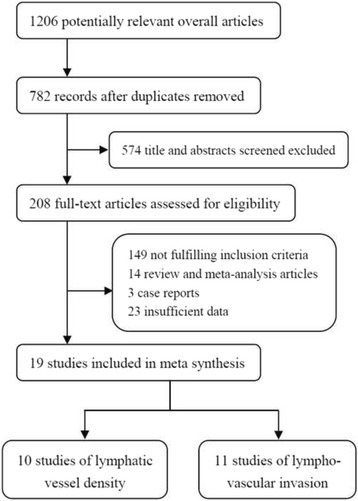
Flow chart of selection of studies for inclusion in meta-analysis

The literature search result is shown in the flow chart of Fig. 1. We initially identified 1206 potential relevant studies from the databases of PubMed, Embase and Web of Science. After removing the duplicated and irrelevant publications, 208 full-text papers were left over. According to the pre-established inclusion criteria, another 189 papers were excluded because of inappropriate publication types, improper staining methods, or insufficient data. Finally, 19 articles were included within this study.

### Characteristics of the included studies

The details of the included 19 studies are exhibited in Tables 1 and 2. A total of 4215 breast cancer patients, aging from 23 to 90 (except one study did not indicate the age [13]), were adopted in this study. Different antibodies, including LYVE-1 in one study, podoplanin in four studies, and D2–40 in 14 studies, were used to label the lymphatic vessels. Lymphatic vessel density was determined by counting the number of lymphatic vessels per area at a variable magnification field under a microscope. Lymphovascular invasion was defined as the presence of tumor emboli within a lymphatic vessel lumen, which was detected by immunohistochemical staining rather than H&E staining. DFS was mentioned as the period from the end of primary treatment until any recurrence occurred. OS was defined as the period from primary surgery until the death of patient.

**Table 1:** Main characteristics and results of the studies evaluating lymphatic microvessel density prognostic values

Author, Year, Country	Size	Age (mean/median, range)	Antibody dilution	Follow-up (month) (mean/median, range)	Cutoff of lymphatic microvessel density	Results
Abe, 2016, Japan [24]	91	54^mean^ (30–81)	D2–40 (1:100)	120^median^ (8–179)	mean	DFS (+), OS (+)
Bono, 2004, UK [17]	180	57^median^ (34–89)	LYVE-1 1 μg/mL	121.2^median^	median	DFS (+), OS (−)
Gu, 2008, China [19]	61	57.59^mean^ (29–90)	podoplanin (1:25)	48.8^mean^	median	DFS (+), OS (+)
Mohammed, 2009, UK [21]	177	57^median^ (32–70)	D2–40 (1:100)	96^median^ (2–184)	median	DFS (+), OS (+)
Mylona, 2007, Greece [5]	109	56.89^mean^ (25–86)	D2–40 (1:20)	96.7^mean^ (5–135)	median	DFS (+), OS (+)
Nakamura, 2005, Japan [6]	113	51^median^ (24–87)	podoplanin (1:200)	116^median^ (10–230)	10/mm^2^	DFS (+), OS (+)
Tsutsui, 2010, Japan [22]	242	58.1^mean^ (23–86)	D2–40 (1:50)	80.64^median^	10.67/field	DFS (+), OS (−)
van der Schaft, 2007, Netherlands [18]	121	61.4^mean^	Podoplanin (not given)	80.5^mean^	median	DFS (+), OS (−)
Zhang, 2008, China [20]	70	49^median^ (30–77)	D2–40 (1:100)	68^median^ (28–83)	median	DFS (+), OS (+)
Zhao, 2012, China [23]	73	53.8^mean^ (29–75)	D2–40 (1:25)	55^mean^ (8–73)	median	DFS (+), OS (+)

*DFS* disease-free survival, *OS* overall survival

**Table 2:** Main characteristics and results of the studies evaluating lymphovascular invasion prognostic values

Author, Year, Country	Size	Age (mean/median, range)	Antibody dilution	Follow-up (month) (mean/median, range)	Positive lymphovascular invasion (%)	Results
Arnaout-Alkarain, 2007, Canada [26]	303	55.5^mean^ (26.6–89.7)	D2–40 (0.1 μg/ml)	91.2^median^	82/303 (27.1)	DFS (+), OS (+)
El-Gohary, 2008, USA [30]	48	64^mean^ (27–89)	D2–40 (1:50)	DFS 30.6^mean^ (12–58) OS 55.2^mean^ (7–84)	18/48 (37.5)	DFS (+), OS (+)
Gudlaugsson, 2011, Norway [13]	240	not given	D2–40 (1:200)	117^median^ (12–192)	51/240 (21.3)	DFS (−), OS (+)
Ito, 2007, Japan [27]	69	52.1^mean^ (27–80)	D2–40 (1:200)	47.5^mean^	16/69 (23.2)	DFS (+), OS (−)
Mohammed, 2011, UK [31]	1005	54^median^ (18–75)	D2–40 (1:100)	107.12^mean^ (1–311)	213/1005 (21.2)	DFS (+), OS (+)
Mohammed, 2014, UK [14]	557	52^median^ (18–72)	D2–40 (1:100)	117^mean^ (4–246)	262/557 (47.0)	DFS (+), OS (+)
Schoppmann, 2004, Austria [25]	374	57.6^median^	podoplanin (1:200)	268.4^mean^ (8–510)	105/374 (28.1)	DFS (+), OS (+)
Tezuka, 2007, Japan [28]	132	55.9^median^ (31–84)	D2–40 (NG)	69^mean^	55/132 (41.7)	DFS (+), OS (−)
van der Schaft, 2007, Netherlands [18]	121	61.4^mean^	Podoplanin (NG)	80.5^mean^	not given	DFS (+), OS (−)
Yamauchi, 2007, Japan [29]	151	53^mean^ (28–84)	D2–40 (1:200)	101^median^	not given	DFS (+), OS (+)
Zhang, 2008, China [20]	70	49^median^ (30–77)	D2–40 (1:100)	68^median^ (28–83)	25/70 (35.7)	DFS (+), OS (−)

*DFS* disease-free survival, *OS* overall survival

### Data analysis

**Fig. 2:**
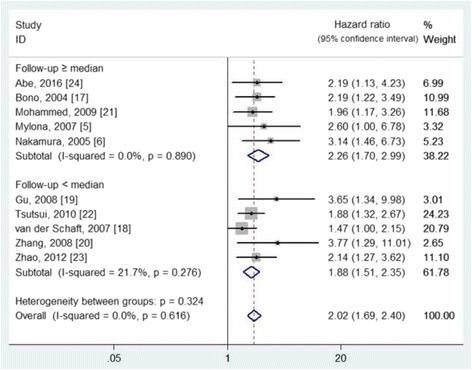
The effect of high lymphatic vessel density on the disease-free survival of patients with primary breast cancer

**Fig. 3:**
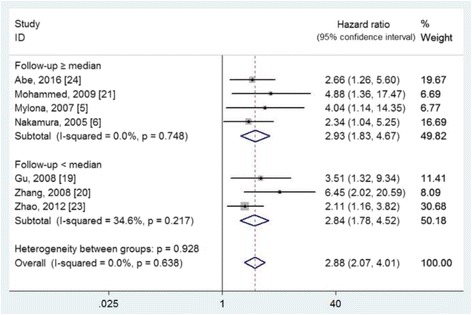
The effect of high lymphatic vessel density on the overall survival of patients with primary breast cancer

Ten studies [5, 6, 17–24], involving 1336 patients (sample sizes ranged from 61 to 242), provided sufficient data to evaluate the effects of lymphatic vessel density on DFS and/or OS (Table 1). The lymphatic vessel density of each study was divided into low and high according to the cutoff value. However, the adopted studies have applied different cutoff values, including the median value in seven studies [5, 17–21, 23], the mean value in one study [24], and the actual value in two studies [6, 22]. The effects of lymphatic vessel density on DFS and OS were assessed in ten and seven studies, with the pooled HR of 2.02 (95% CIs 1.69 to 2.40, I^2^ = 0.0%, *P* = 0.616) for DFS (Fig. 2) and 2.88 (95% CIs 2.07 to 4.01, I^2^ = 0.0%, *P* = 0.638) for OS (Fig. 3), respectively. According to the median value of follow-up period, the included studies were divided into two subgroups of ≥ median and < median. The detailed results are shown in Figs. 2 and 3.

**Fig. 4:**
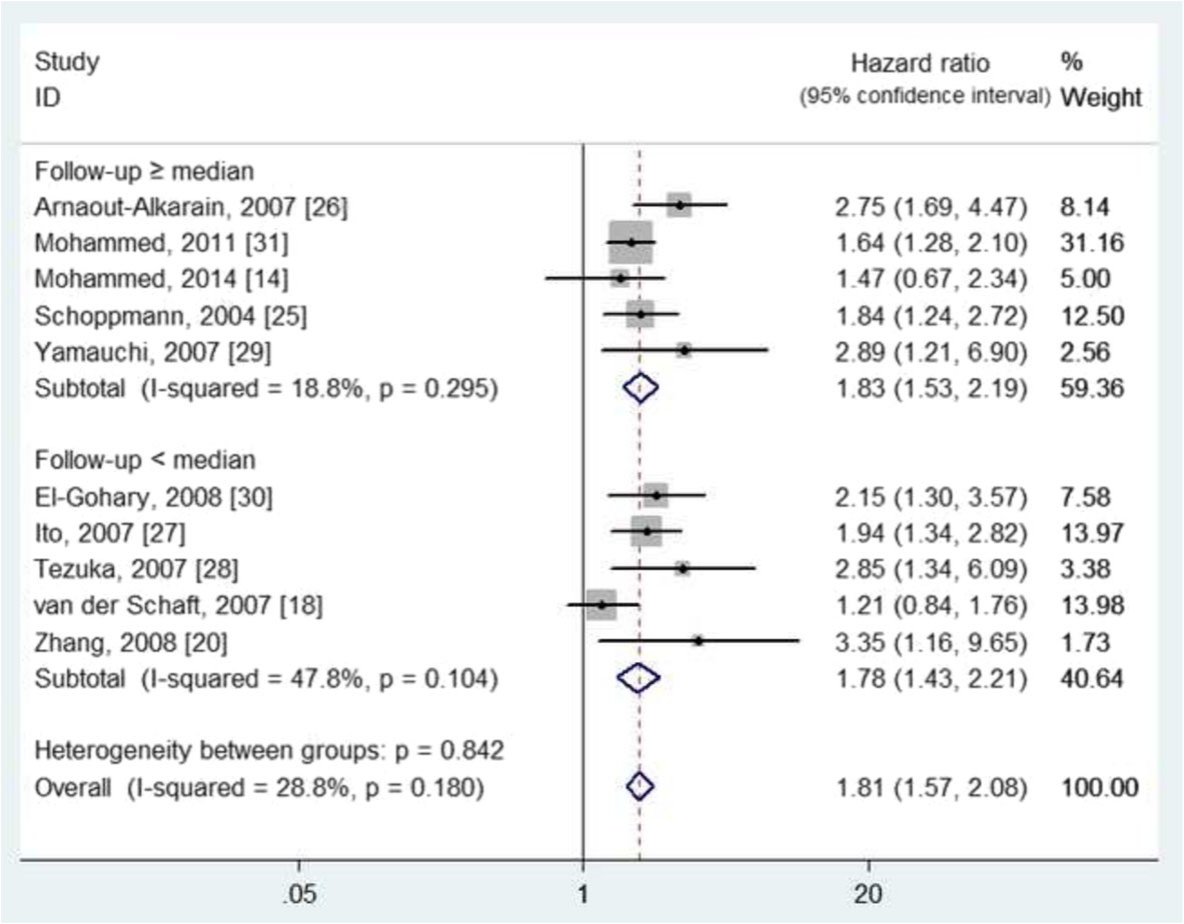
The effect of lymphovascular invasion presence on the disease-free survival of patients with primary breast cancer

**Fig. 5:**
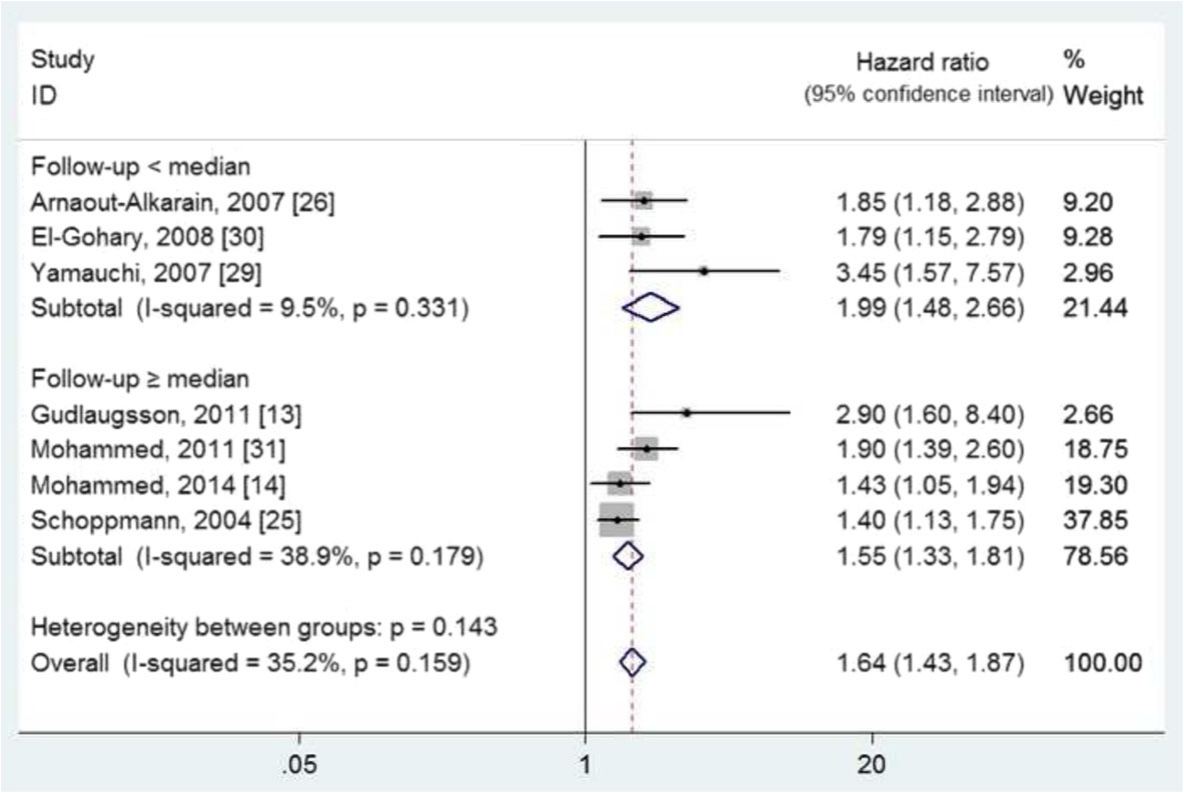
The effect of lymphovascular invasion presence on the overall survival of patients with primary breast cancer

On the other hand, 11studies [13, 14, 18, 20, 25–31], involving 3070 patients (sample sizes ranged from 48 to1005), were eligible to evaluate the prognostic value of lymphovascular invasion (Table 2). All of the included studies used the presence of lymphovascular invasion to evaluate its prognostic value. It means that the cutoff value is defined as the presence or not of lymphovascular invasion. And nine of them reported the detection rate of lymphovascular invasion in breast cancer [13, 14, 20, 25–28, 30, 31]. The detection rates were ranged from 21.2 to 47.0%, with an overall detection rate of 29.56% (827/2798). The effect of lymphovascular invasion on DFS and OS was evaluated in ten and seven studies, respectively. The pooled HRs were 1.81 (95% CIs 1.57 to 2.08, I^2^ = 28.8%, *P* = 0.180) for DFS (Fig. 4) and 1.64 (95% CIs 1.43 to 1.87, I^2^ = 35.2%, *P* = 0.159) for OS (Fig. 5), with no evidence of heterogeneity. According to the median value of follow-up period, the included studies were also divided into two subgroups of ≥ median and < median, which showed no heterogeneity (*P* > 0.05) (Figs. 4 and 5).

### Sensitivity analysis and publication bias

In order to assess the stability of the results, sensitivity analyses were independently performed in lymphatic vessel density group and lymphovascular invasion group. By removing one study sequentially, sensitivity analyses yielded consistent results, indicating statistically robust results of the analyses (Additional file 1: Figure S1 ). Begg’s tests and the funnel plots of the HR values against the standard error of HR values showed no substantial asymmetry (Additional file 1: Figure S2). There was no evidence of publication bias exhibited in the Egger’s regression test.

## Discussion

The current meta-analysis study indicates that both lymphatic vessel density and lymphovascular invasion presence can predict poor prognosis in females with breast cancer. Compared with the high lymphatic vessel density, the presence of lymphovascular invasion in breast cancer appears to have weaker impacts on DFS and OS; but it is also significantly associated with poor survival. Furthermore, lymphovascular invasion was present in 29.56% of breast cancer patients, who would have poorer prognosis.

The metastasis routes of breast cancer consist of local invasion, hematogenous metastasis, and lymphatic metastasis. New blood and lymphatic vessels formed through physiological or pathological processes are called angiogenesis and lymphangiogenesis, respectively. It is well known that tumor angiogenesis, and its indicator blood vessel density are closely associated with the clinicopathological outcomes of breast cancer [32]. A meta-analysis study performed by Uzzan et al. has shown that the high blood vessel density can predict poor survival in breast cancer (risk ratio = 1.54 for DFS and OS with the same 95% CI 1.29–1.84) [9]. However, the prognostic value of lymphatic vessel density is still uncertain [33]. With the development of lymphatic vessel biology, lymphatic vascular system has been considered as an active player involved in breast cancer [34]. Our meta-analysis result shows that high lymphatic vessel density has unfavorable impacts on DFS (HR 2.02, 95% CI 1.69 to 2.40) and OS (HR 2.88, 95% CI 2.07 to 4.01). Compare with blood vessel density, lymphatic vessel density even displays a stronger predictive value in breast cancer.

The result that lymphatic vessel density is a risk factor of poor survival is supported by all included studies; however, the values of lymphatic vessel density were differentiated notably in these studies [17, 21, 24]. The variation might be caused by patient sources, staining techniques, antibody categories and antibody dilutions. In addition, different counting methods of lymphatic vessel density, by using different hotspots (three [19], four [18], and five [24]), magnification field (100× [22], 200× [24], 400× [19]), and measuring unit (vessels/mm2 [24], vessels/area [22]), are also accounted for the variation of results. Furthermore, the cutoff value to divide lymphatic vessel density as low and high is a crucial factor that cannot be ignored. Because the asset value of lymphatic vessel density is not a normal distribution, seven in ten studies chose the median value as the cutoff value, other three studies took the mean or actual value as the cutoff value. Therefore, studies with more standardized and stricter design are required in the assessment of lymphatic vessel density.

Due to lack of the specific markers of lymphatic endothelium cells, most of the previous studies have detected lymphovascular invasion using H&E staining method [11, 12]. One major challenge of this method is to distinguish lymphovascular invasion from retraction artifacts caused by tissue handling and fixation on H&E stained sections. Another challenge is that lymphovascular invasion may be missed if tumor cells are packed in a small vessel [35]. With the help of specific markers, such as D2–40/podoplanin, LYVE-1, VEGFR-3, and Prox-1, lymphatic vessels can be effectively distinguished from blood vessels or retraction artifacts. A previous study has compared the reliability of immunohistochemical staining with that of H&E staining [36]. The results showed that the detection rate of lymphovascular invasion widely ranged from 10 to 49% for H&E staining; however, the range was narrower using immunohistochemical staining (ranged from 21 to 42%) [36]. It indicates that immunohistochemical staining should be more reliable for identifying lymphovascular invasion. Therefore, we conducted a meta-analysis to study the prognostic value of lymphovascular invasion, which was assessed by immunohistochemical staining instead of H&E staining [35].

With the accumulating evidence, we conducted an update meta-analysis study to re-evaluate the prognostic value of lymphovascular invasion. The result shows that lymphovascular invasion, detected by immunohistochemical staining, has an unfavorable impact on survival, in line with the previous study [36]. However, the result should be analysed more thoroughly. Mohammed et al. [14, 31] has demonstrated that the impact of lymphovascular invasion is mainly found in breast cancer patients with negative lymph node metastasis and with a single positive lymph node metastasis. Moreover, the frequency of lymphovascular invasion per tumor lesion has no effects on prognosis in lymph node negative and lymph node positive patients [14, 31]. Besides, the location of lymphovascular invasion [23, 30] and the patients’ age [25] also have influence on the survival of breast cancer patients.

The current meta-analysis study has some strengths. The results show that both lymphatic vessel density and lymphovascular invasion are unfavorable predictors on DFS and OS in breast cancer. The included 19 studies and 4215 participants enhanced the statistical power and provided more reliable results. However, some limitations should be considered. All included studies were observational studies with relatively small sample sizes. Selection bias and recall bias were inevitable. Besides, the values of lymphatic vessel density varied notably due to unmeasured or inadequately measured factors. It resulted that different cutoff values were used to define high and low lymphatic vessel density subgroups in different studies. Although there are no heterogeneities show in each subgroup, the deviations caused by different cutoff values cannot be ignored. Nevertheless, the conclusion that higher lymphatic vessel density is associated with poor survival is reasonable even with different cutoff values. Therefore, strictly controlled studies with larger sample sizes are needed.

## Conclusions

The study demonstrates that the high lymphatic vessel density and the presence of lymphovascular invasion both are unfavorable prognostic factors in primary breast cancer. Compared with lymphatic vessel density, lymphovascular invasion shows a weaker influence on patients’ survival, but it is also an important risk factor in breast cancer. Counting methods of lymphatic vessel density, choice of appropriate cutoff value, thoroughly analysis of lymphovascular invasion, and standardized design of study, are the crucial points need to be considered.

## Additional file

Additional file 1: Figure S1. Sensitivity analysis of the included studies reporting the prognostic values of lymphatic vessel density and lymphovascular invasion. **Figure S2.** Begg’s funnel plot of the included studies reporting the prognostic values of lymphatic vessel density and lymphovascular invasion. (PDF 430 kb)

## Abbreviations

CI: Confidence interval
DFS: Disease-free survival
H&E: Hematoxylin and eosin
HR: Hazard ratio
OS: Overall survival
