# Supplementary material for: High lymphatic vessel density and presence of lymphovascular invasion both predict poor prognosis in breast cancer
Source: BMC Cancer. 2017 May 17;17:335. doi: 10.1186/s12885-017-3338-x (PMC5436442; doi:10.1186/s12885-017-3338-x)
Supplement: Additional file 1: Figure S1. — Sensitivity analysis of the included studies reporting the prognostic values of lymphatic vessel density and lymphovascular invasion. Figure S2. Begg’s funnel plot of the included studies reporting the prognostic values of lymphatic vessel density and lymphovascular invasion. (PDF 430 kb) [file 12885_2017_3338_MOESM1_ESM.pdf]

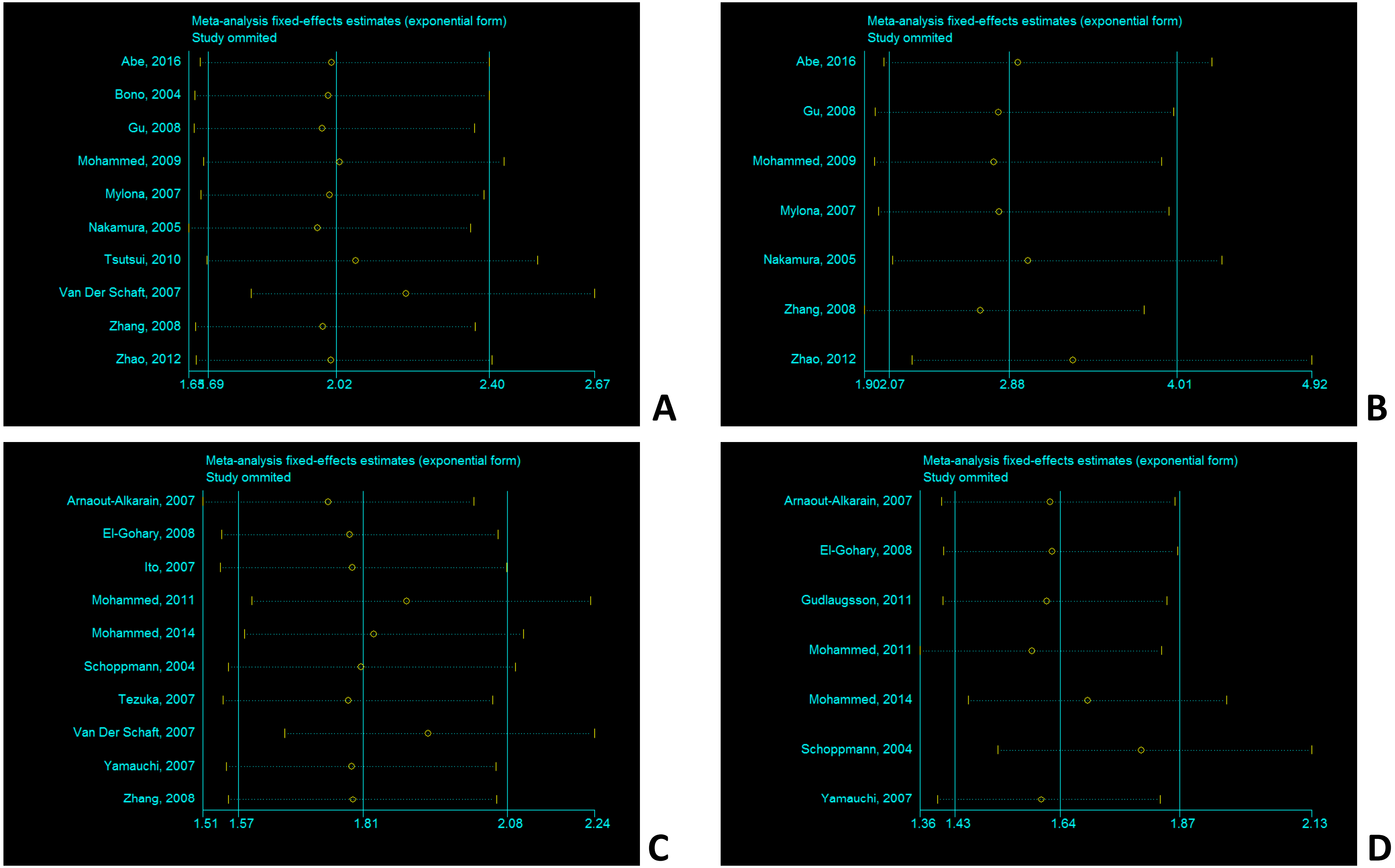


**Figure S1:** Sensitivity analysis of the included studies reporting the prognostic values of lymphatic vessel density and lymphovascular invasion.

**A.** Lymphatic vessel density with disease-free survival; **B.** lymphatic vessel density with overall survival; **C.** lymphovascular invasion with disease-free survival; **D.** lymphovascular invasion with overall survival.

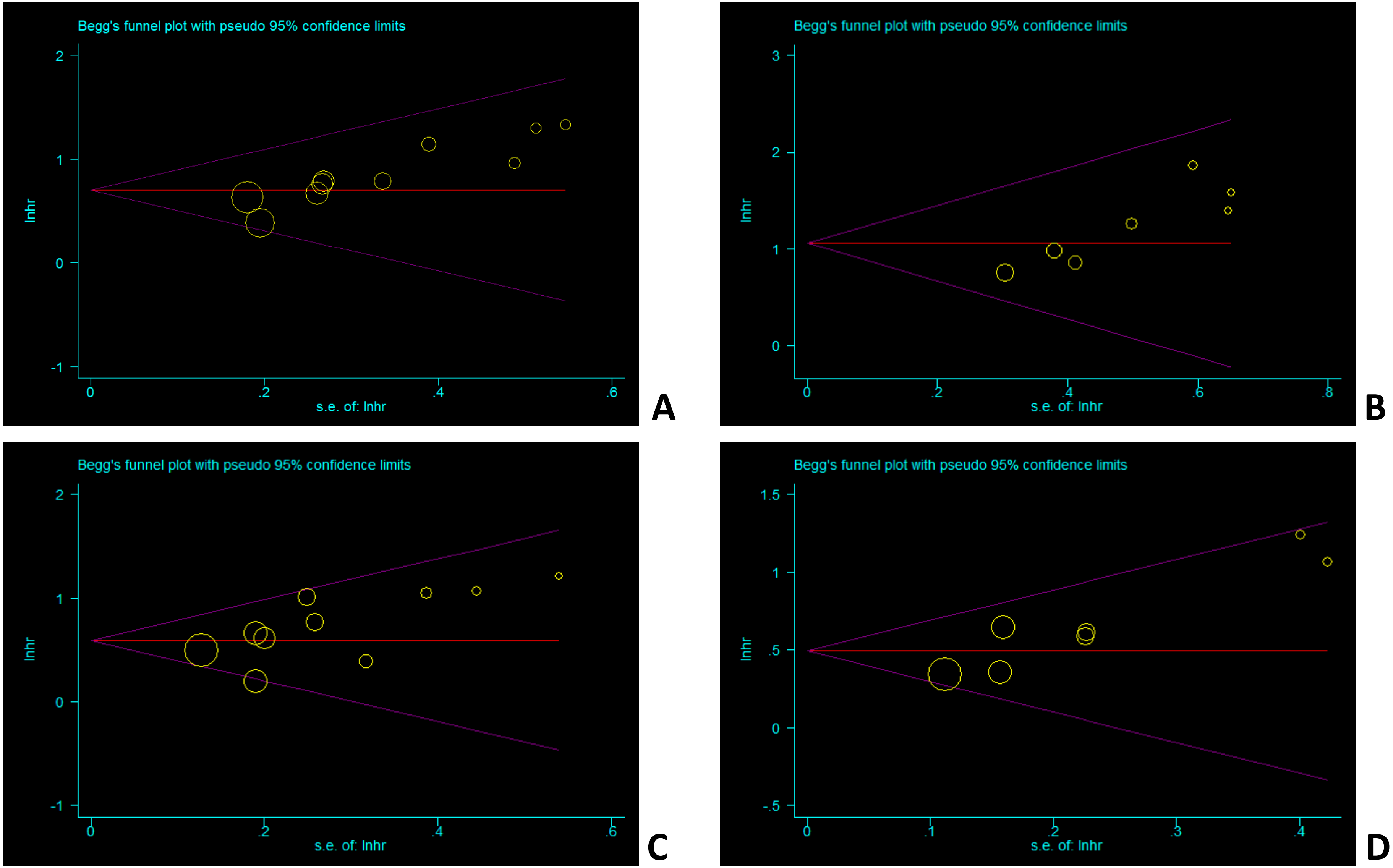


**Figure S2:** Begg's funnel plot of the included studies reporting the prognostic values of lymphatic vessel density and lymphovascular invasion.

**A.** lymphatic vessel density with disease-free survival; **B.** lymphatic vessel density with overall survival; **C.** lymphovascular invasion with disease-free survival; **D.** lymphovascular invasion with overall survival.
